# Autophagy inhibition specifically promotes epithelial-mesenchymal transition and invasion in RAS-mutated cancer cells

**DOI:** 10.1080/15548627.2019.1569912

**Published:** 2019-02-20

**Authors:** Yihua Wang, Hua Xiong, Dian Liu, Charlotte Hill, Ayse Ertay, Juanjuan Li, Yanmei Zou, Paul Miller, Eileen White, Julian Downward, Robert D Goldin, Xianglin Yuan, Xin Lu

**Affiliations:** a Department of Oncology, Tongji Hospital, Tongji Medical College, Huazhong University of Science and Technology, Wuhan, China; b Biological Sciences, Faculty of Environmental and Life Sciences, University of Southampton, Southampton, UK; c Institute for Life Sciences, University of Southampton, Southampton, UK; d Ludwig Institute for Cancer Research Ltd., Nuffield Department of Clinical Medicine, University of Oxford, Oxford, UK; e Rutgers Cancer Institute of New Jersey, New Brunswick, NJ, USA; f Oncogene Biology Laboratory, The Francis Crick Institute, London, UK; g Centre for Pathology, St Mary’s Hospital, Imperial College London, London, UK

**Keywords:** Autophagy, EMT, NFKB/NF-0κB, RAS, SQSTM1/p62

## Abstract

Macroautophagy/autophagy inhibition is a novel anticancer therapeutic strategy, especially for tumors driven by mutant *RAS*. Here, we demonstrate that autophagy inhibition in *RAS*-mutated cells induces epithelial-mesenchymal transition (EMT), which is associated with enhanced tumor invasion. This is at least partially achieved by triggering the NFKB/NF-κB pathway via SQSTM1/p62. Knockdown of *ATG3* or *ATG5* increases oncogenic *RAS*-induced expression of ZEB1 and SNAI2/Snail2, and activates NFKB activity. Depletion of *SQSTM1* abolishes the activation of the NFKB pathway induced by autophagy inhibition in *RAS*-mutated cells. NFKB pathway inhibition by depletion of *RELA*/p65 blocks this EMT induction. Finally, accumulation of SQSTM1 protein correlates with loss of CDH1/E-cadherin expression in pancreatic adenocarcinoma. Together, we suggest that combining autophagy inhibition with NFKB inhibitors may therefore be necessary to treat *RAS*-mutated cancer.

**Abbreviations:** 4-OHT: 4-hydroxytamoxifen; DIC: differential interference contrast; EMT: epithelial-mesenchymal transition; ESR: estrogen receptor; MAPK/ERK: mitogen-activated protein kinase; iBMK: immortalized baby mouse kidney epithelial cells; MET: mesenchymal-epithelial transition; PI3K: phosphoinositide 3-kinase; RNAi: RNA interference; TGFB/TGF-β: transforming growth factor beta; TNF: tumor necrosis factor; TRAF6: TNF receptor associated factor 6.

## Introduction

Autophagy is an evolutionarily conserved biological process that degrades long-lived proteins and cytoplasmic organelles. The manipulation of autophagy has emerged as a new therapeutic strategy with which to treat neurodegenerative diseases and cancer [1–3]. Autophagy has a double-edged sword effect in cancer [2,3]. Autophagy’s tumor suppressive function is partly attributed to its ability to induce cell death via several mechanisms [4,5]; however, a positive role of autophagy in tumorigenesis has also been established. Emerging studies have shown that the RAS signalling pathway is one of the oncogenic pathways influenced by autophagic activity [6–12]. Oncogenic *RAS* mutations, which activate both the RAF-MAPK/ERK (mitogen-activated protein kinase) and phosphoinositide 3-kinase (PI3K)-AKT signalling pathways, are among the most prevalent genetic changes found in human cancers, occurring in approximately 20% of human tumors [13,14]. The mutation rate of *RAS* varies depending on tumor type, with *KRAS* mutations occurring in over 90% of pancreatic carcinomas and 30–50% of colorectal cancer. Yang and colleagues demonstrated that the inhibition of autophagy in pancreatic tumor cells suppressed their growth by reducing the production of reactive oxygen species, thus limiting effective metabolism via decreased mitochondrial oxidative phosphorylation and increased DNA damage [8]. This finding provides an example of a positive role of autophagy in pancreatic tumorigenesis [8]. Accordingly, there are several phase I/II clinical trials in progress using the autophagy inhibitors chloroquine or hydroxychloroquine in combination with chemotherapy for the treatment of a range of tumors, including pancreatic cancer [15].

Although the rationale for such studies is supported by strong preclinical data, many open questions and controversies remain regarding autophagy as a target in cancer therapy [16]. Some potential caveats associated with autophagy inhibition in cancer therapy warrant consideration. There are concerns about whether autophagy inhibition treatment may increase the incidence of tumor invasion and metastasis. In order to invade, disseminate to distant tissues and subsequently form metastatic colonies, neoplastic epithelial cells, which exhibit predominantly epithelial cancer cell phenotype, must shift, at least transiently, into a more mesenchymal cancer cell phenotype. This shift is achieved by the activation of the complex cell-biological program termed the epithelial-mesenchymal transition (EMT) [17], which is a cellular reprogramming process that is mainly induced by a number of transcription factors, such as SNAIs/Snails, TWISTs and ZEBs, that bind E-boxes in the proximal promoter of the *CDH1*/E-cadherin gene and repress its expression [18]. Currently, there are controversial reports available regarding the effect of autophagy on the regulation of EMT [19], and it is likely dependent on the cellular type and/or on stage of tumor progression.

Several works show that defects in the autophagic machinery restrain dissemination and metastatic spreading of cancer [20–22]. In line with these facts, inhibition of autophagy reduces FSTL1 (follistatin like 1)-induced EMT in human bronchial epithelial cells [23], whereas ATG4A overexpression significantly promotes EMT in gastric cancer cells [24]. Conversely, there is evidence indicating that autophagy acts to prevent EMT and that the activation of the autophagic machinery may determine reversion of the EMT phenotype in cancer cells [25–34]. Thus, understanding the factors that determine the effect of autophagy on EMT is crucial before we apply autophagy inhibitors or inducers to treat cancer patients. Here, we report that autophagy inhibition specifically promotes EMT and invasion in *RAS*-mutated cancer cells, but not in *RAS* wild-type cells. This is achieved, at least partially, by an elevation in SQSTM1/p62 expression that induces RELA/p65 mediated-transactivation of EMT transcription factors such as ZEB1 and SNAI2/Snail2.

## Results

### Autophagy inhibition specifically activates the EMT program in RAS-mutated cancer cells

**Figure 1.**
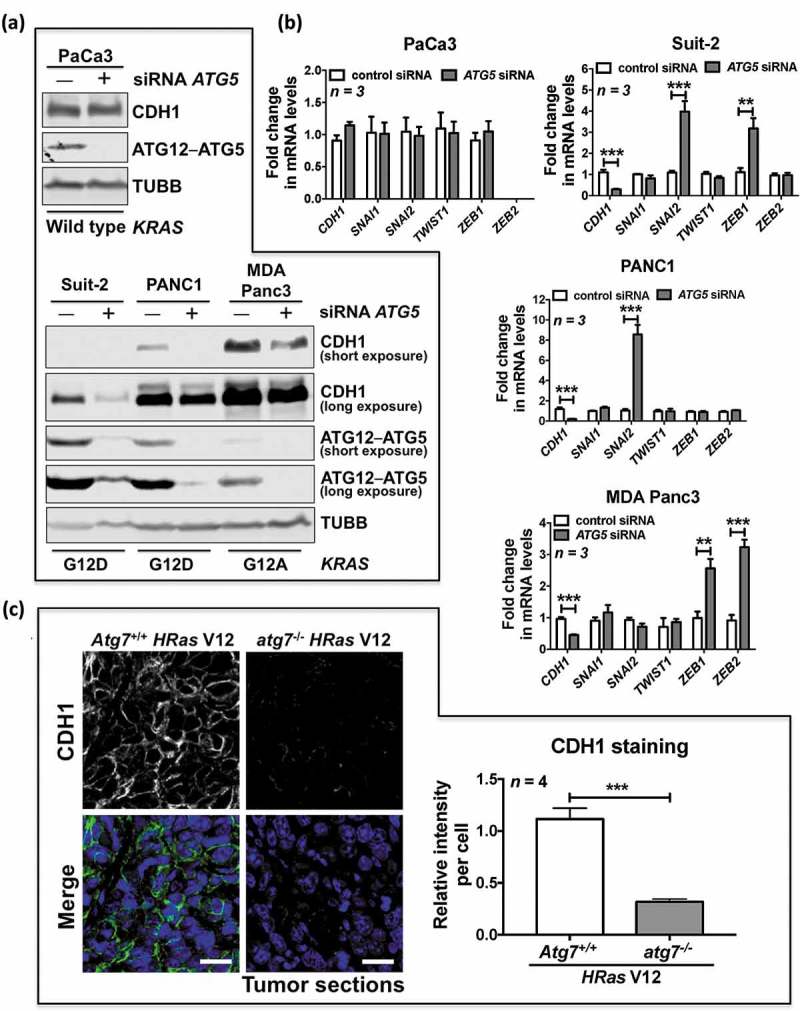
Autophagy inhibition promotes EMT in *RAS*-mutated cells. (a) Protein expression of CDH1 and ATG12–ATG5 in the indicated pancreatic cancer cell lines transfected with control siRNA or *ATG5* siRNA. TUBB/β1-tubulin was used as a loading control. For protein expression of CDH1 and ATG12–ATG5 in pancreatic cancer cell lines with mutant *KRAS*, both short and long exposures (respectively) are shown. *KRAS* mutation status is indicated under the blots. (b) Fold change in mRNA levels of *CDH1, SNAI1, SNAI2, TWIST1, ZEB1* and *ZEB2* in the indicated pancreatic cancer cell lines transfected with control siRNA or *ATG5* siRNA. *GAPDH*-normalized mRNA levels in control cells were used to set the baseline value at unity. Data are mean ± s.d. *n* = 3 samples per group. * *P* < 0.05. ** *P* < 0.01. *** *P* < 0.001. (c) Immunofluorescence staining of CDH1 (green) in *HRas* V12-expressing *Atg7*^+/+^ tumors or *HRas* V12-expressing *atg7*^−/-^ tumors. TO-PRO-3 (blue) was used to stain nucleic acids. Scale bar: 20 μm. *Atg7*^+/+^ or *atg7*^−/-^ iBMK cells transduced with *HRas* V12 were subcutaneously injected in nude mice to form tumors. The graph shows the average relative intensity of CDH1 per cell evaluated using ImageJ, and data are mean ± s.d. *n* = 4 random fields. *** *P* < 0.001.

To investigate whether *RAS* mutational status influences the effect of autophagy in regulating EMT, we used RNA interference (RNAi) to deplete *ATG5*, an important component of the autophagic machinery, in 7 human cancer cell lines derived from the pancreas (PaCa3, Suit-2, PANC1 and MDA Panc3) and colon (HCT116, HKe3 and HKh2). Four of these cancer cell lines express mutant *KRAS* (Suit-2, PANC1, MDA Panc3 and HCT116) [35], whereas PaCa3, HKe3 and HKh2 lines express wild-type *RAS. ATG5* depletion led to a clear reduction in CDH1 protein and mRNA expression in all cancer cell lines that express mutant *KRAS*, including Suit-2 (*KRAS* G12D), PANC1 (*KRAS* G12D), MDA Panc3 (*KRAS* G12A), and HCT116 (*KRAS* G13D) (Figure 1(a, b); Figure S1(a, b). Remarkably, under the same conditions, *ATG5* knockdown had no effect on CDH1 expression in all 3 wild-type *RAS* expressing cell lines, including PaCa3, HKe3 and HKh2 (Figure 1(a, b); Figure S1(a)). Importantly, the HKe3 and HKh2 lines are isogenic counterparts of HCT116, in which the allele of *KRAS* G13D is disrupted by homologous recombination [35]. Thus, there is only one allele of wild-type *KRAS* in the HKe3 and HKh2 lines.

EMT is a cellular reprogramming process that is mainly induced by a number of transcription factors, such as SNAI1/Snail1, SNAI2, TWIST1, ZEB1 and ZEB2, which bind E-boxes in the proximal promoter of the *CDH1* gene to repress its expression [18]. We thus investigated the impact of *ATG5* RNAi on the expression levels of EMT transcription factors in the same panel of cancer cell lines. In wild-type *RAS*-expressing PaCa3 cells, there were no significant inductions of EMT transcription factors (Figure 1(b)). However, in the cells with mutant *RAS*, the EMT transcription factors were induced with different expression patterns. Following *ATG5* depletion, we observed upregulation of *ZEB1* and *SNAI2* in Suit-2 and HCT116, upregulation of *SNAI2* in PANC1, and upregulation of *ZEB1* and *ZEB2* in MDA Panc3 (Figure 1(b); Figure S1(b)).

When grown in nude mice, nontumorigenic baby mouse kidney epithelial (iBMK) cells transduced with *HRas* V12 form tumors [10]. Although, as shown previously [10], oncogenic *RAS*-expressing *atg5*^−/-^ or *atg7*^−/-^ cells display reduced tumor growth, we found that CDH1 expression was largely reduced in oncogenic *RAS*-expressing *atg7*^−/-^ or *atg5*^−/-^ tumors compared to oncogenic *RAS*-expressing *Atg7*^+/+^ or *Atg5*^+/+^ tumors (Figure 1(c); Figure S1(c)). These data suggest that EMT is induced in autophagy-deficient *RAS*-mutated tumors *in vivo*.

Together, these results demonstrate that autophagy inhibition is able to activate the EMT program, specifically in *RAS*-mutated cancer cells, which is supported by the induction of EMT transcription factors and, consequently, a reduction in CDH1 expression.

### Autophagy inhibition cooperates with RAS activation to induce EMT

To further confirm the observation above, we utilized the established RAS-induced EMT model [36,37]. HKe3 ER:HRAS V12 cells were generated by introducing a regulatable RAS construct into HKe3 cells comprising mutant *HRAS* fused to the ESR (estrogen receptor) ligand-binding domain that is conditionally responsive to 4-hydroxytamoxifen (OHT). Addition of 4-OHT acutely activates the RAS pathway in HKe-3 cells expressing ER:HRAS V12 and induces EMT [36,37].

**Figure 2.**
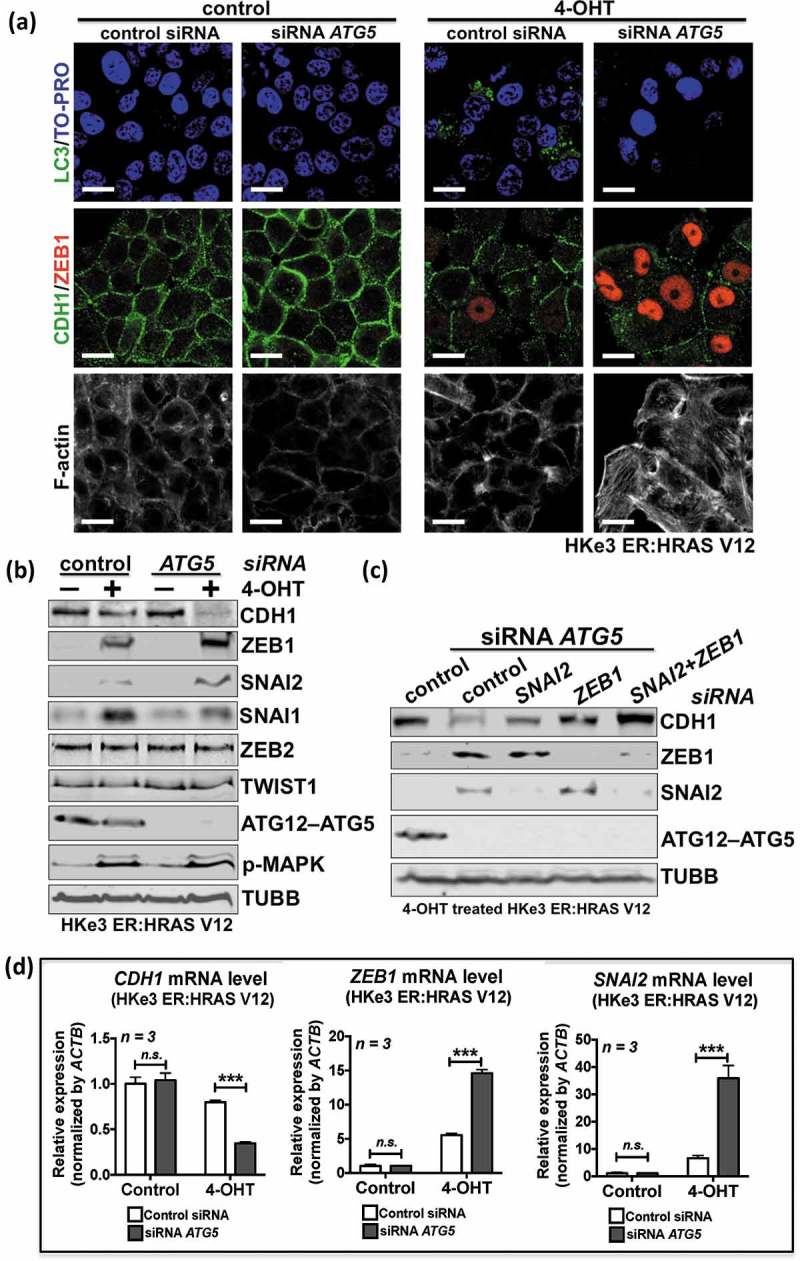
Autophagy inhibition promotes oncogenic *RAS*-induced EMT. (a) Immunofluorescence staining of LC3 (green), CDH1 (green), ZEB1 (red) or F-actin in HKe3 ER:HRAS V12 cells with the indicated treatments. Rhodamine-phalloidin was used to stain F-actin. TO-PRO-3 (blue) was used to stain nucleic acids. Scale bar: 20μm. (b) Protein expression of CDH1, ZEB1, SNAI2, SNAI1, ZEB2, TWIST1, ATG12–ATG5 and phospho-MAPK/ERK (p-MAPK) in HKe3 ER:HRAS V12 cells with the indicated treatments. TUBB was used as a loading control. (c) Fold change in mRNA levels of *CDH1, ZEB1* and *SNAI2* in HKe3 ER:HRAS V12 cells with the indicated treatments. mRNA levels normalized to *ACTB*/β-actin in control cells were used to set the baseline value at unity. Data are mean ± s.d. *n* = 3 samples per group. *n.s. P* > 0.05, *** *P* < 0.001. (d) Protein expression of CDH1, ZEB1, SNAI2 and ATG12–ATG5 in 4-OHT-treated HKe3 ER:HRAS V12 cells with the indicated treatments. TUBB was used as a loading control.

Oncogenic *RAS* activation induced autophagic activity, as demonstrated by MAP1LC3/LC3 puncta staining (Figure 2(a)) and an increase in LC3-II by western blot analysis (Fig. S2A). Knockdown of *ATG5* blocked the autophagic activation induced by oncogenic *RAS* (Figure 2(a); Figure S2(a)). We have shown previously that oncogenic *RAS* activation leads to EMT in these cells [36,37] (Figure 2). Interestingly, *ATG5* knockdown together with oncogenic *RAS* activation achieved a synergistic effect in inducing EMT, reflected by a larger increase in ZEB1 expression and a further reduction in CDH1 levels, as well as a replacement of cortical actin filaments by actin stress fibers and a scattered cellular phenotype (Figure 2(a), 4-OHT group; Figure 2(b)). As aforementioned (Figure 1(a, b)), depletion of *ATG5* in wild-type *RAS* cells did not significantly induce EMT (Figure 2(a), control group; Figure 2(b)), confirming that autophagy inhibition specifically promotes mutant *RAS*-induced EMT.

To eliminate the autophagy-independent effect of ATG5, we also inhibited autophagic activity by depletion of another key component in the autophagic machinery, ATG3. Knockdown of *ATG3* or *ATG5* blocked oncogenic *RAS*-induced autophagy, as measured by the overall level of LC3-II (Fig. S2A). *ATG5* or *ATG3* knockdown alone in HKe3 ER:HRAS V12 cells slightly increased the mRNA levels of *ZEB1* and *SNAI2*. The addition of 4-OHT to activate mutant *RAS* in HKe3 ER:HRAS V12 cells led to a 10-fold increase of *ZEB1* mRNA levels and a 20-fold increase of *SNAI2* mRNA, which is consistent with our previous report [36,37]. Most strikingly, *ATG5* or *ATG3* knockdown together with oncogenic *RAS* activation achieved a synergistic effect in inducing EMT, reflected by a larger increase in *ZEB1, SNAI2* and *VIM* (vimentin) expression, and a further downregulation of CDH1 (Figure 2(b, c); Figure S2). *ATG5* depletion in 4-OHT-treated HKe3 ER:HRAS V12 cells induced a 20-fold increase in *ZEB1* mRNA, an 80-fold increase in *SNAI2* mRNA and a 40-fold increase in *VIM* mRNA; while *ATG3* depletion in RAS-activated cells induced a 30-fold increase in *ZEB1* mRNA, a 120-fold increase in *SNAI2* mRNA and a 40-fold increase in *VIM* mRNA (Fig. S2B). However, the effects on *SNAI1, ZEB2* and *TWIST1* were minimal (Figure 2(b); Figure S2(b)). In addition, *ZEB1* RNAi or *SNAI2* RNAi alone induced a small but detectable increase of CDH1 protein level in *ATG5*-depleted HKe3–ER:HRASV12 cells treated with 4-OHT (Figure 2(d)). Double knockdown of *SNAI2* and *ZEB1* achieved an additive effect, reflected by a larger increase in CDH1 expression than that observed in cells treated with *SNAI2* or *ZEB1* RNAi alone (Figure 2(d)). These data suggest that autophagy inhibition specifically promotes EMT in RAS-mutated cells, mainly via upregulation of ZEB1 and to a lesser extent via SNAI2.

EMT can also be induced by several extracellular cues in the microenvironment of a given epithelial tissue, such as TGFB/TGF-β (transforming growth factor beta) [38]. We found autophagy inhibition via RNAi-mediated depletion of *ATG5* or *ATG3* did not promote TGFB-induced EMT in HaCat cells (Fig. S3A). We further tested this with another ER:HRAS V12 system in an immortalized human breast epithelial cell line, MCF10A. *ATG5* knockdown together with oncogenic *RAS* activation, but not TGFB, achieved a synergistic effect in inducing EMT, reflected by a further reduction in *CDH1* levels (Fig. S3B). These data indicate that autophagy inhibition specifically promotes RAS-induced, but not TGFB-induced EMT.

### Autophagy inhibition cooperates with RAS activation to promote cell migration and to induce invasion

**Figure 3.**
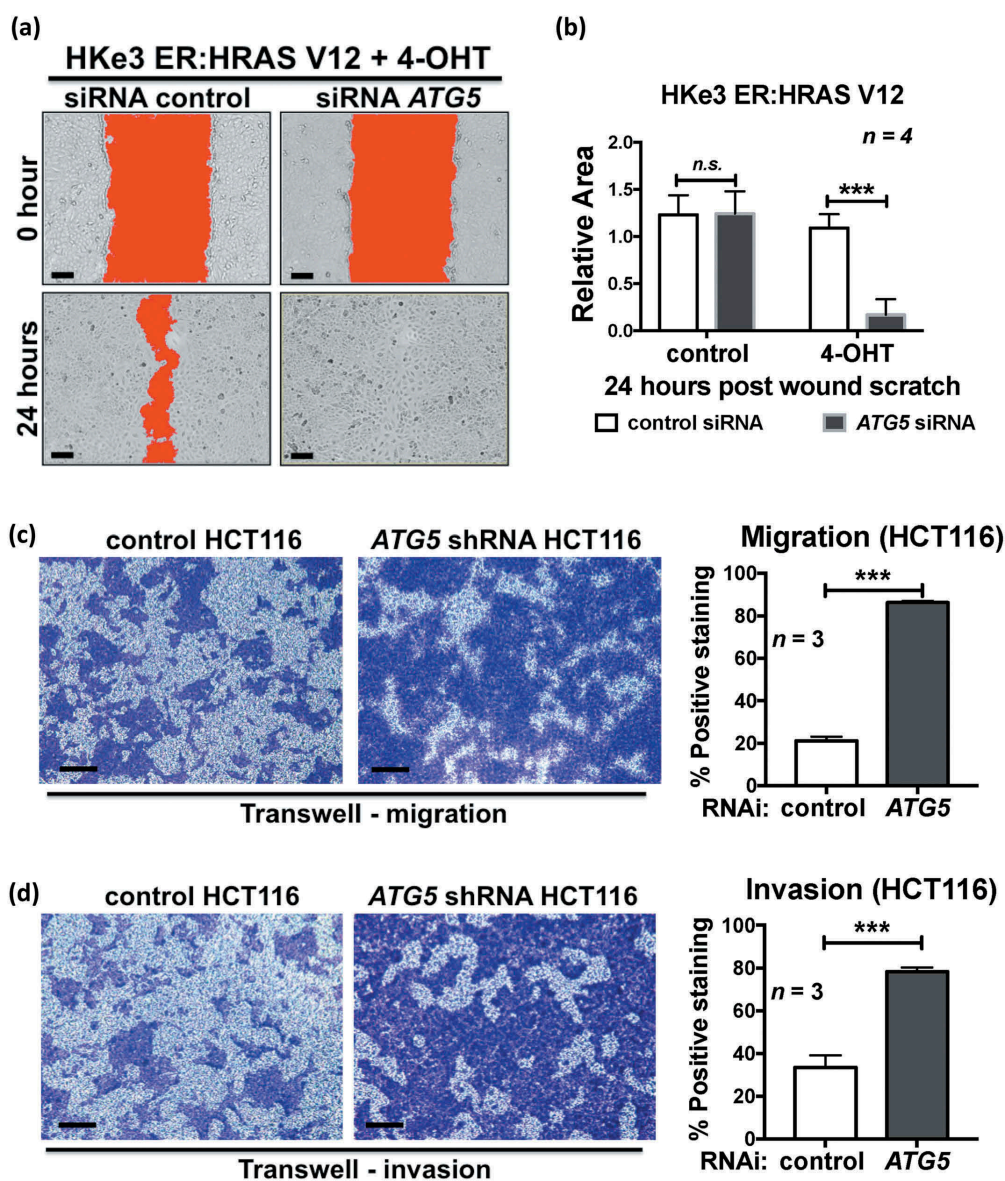
Autophagy inhibition promotes oncogenic *RAS*-induced cell migration and invasion. (a) Scratch wound assay of 4-OHT-treated HKe3 ER:HRAS V12 cells transfected with control or *ATG5* siRNA. Representative images of 4-OHT-treated HKe3 ER:HRAS V12 cells with the indicated treatments at time 0 h or 24 h after the scratch wound. Wounds have been artificially colored red to aid visualization. Scale bar: 50 μm. (b) Scratch wound assay of control or 4-OHT-treated HKe3 ER:HRAS V12 cells transfected with control or *ATG5* siRNA 24 h after the scratch wound. The graph shows the area of a wound evaluated with ImageJ, and data are mean ± s.d. *n* = 4. *n.s. P* > 0.05, *** *P* < 0.001. (c) Transwell migration assays in HCT116 cells infected with control or *ATG5* shRNA. Cells were stained with crystal violet. Scale bar: 100 μm. Data are mean ± s.d. *n* = 3. *** *P* < 0.001. (d) Transwell Matrigel invasion assays in HCT116 cells infected with control or *ATG5* shRNA. Cells were stained with crystal violet. Scale bar: 100 μm. Data are mean ± s.d. *n* = 3. *** *P* < 0.001.

The EMT process allows epithelial cells to detach from the primary site and invade surrounding tissues in order to migrate to and colonize a new site [17]. ATG5’s ability to affect cell mobility was tested using a wound scratch assay. Twenty-four h after the scratch wound, *ATG5* RNAi-transfected 4-OHT-treated HKe3 ER:HRAS V12 cells filled all the gaps, whereas the wound in the control cells was still open (Figure 3(a, b); Fig. S4A). However, under the same conditions, *ATG5* knockdown had no effect on cell migration in control HKe3 ER:HRAS V12 cells, in which mutant *RAS* is not activated (Figure 3(b)). In addition, we stably expressed control or *ATG5* shRNA in HCT116 cells that have mutated *KRAS* G13D (Fig. S4B). In the Transwell migration and Matrigel invasion assays, *ATG5* shRNA promoted both cell migration and invasion in HCT116 cells (Figure 3(c, d)).

To further investigate whether autophagy inhibition cooperates with RAS activation to induce invasion, we tested this with MCF10A ER:HRAS V12 cells, which form highly polarized epithelial acini (3D culture model) when grown on a thick layer of Matrigel. This system is used extensively to study invasion and pathways that can perturb the breast epithelial architecture [37,39]. We adopted this experimental system and used MCF10A ER:HRAS V12 cells transfected with control or *ATG5* siRNA to investigate whether autophagy inhibition promotes oncogenic *RAS*-induced EMT and invasion in 3D cultures (Fig. S4C-E). We recovered these cells from the Matrigel, and examined the protein expression. *ATG5* knockdown in oncogenic *RAS*-activated MCF10A cells, achieved a synergistic effect in inducing EMT in 3D cultures, reflected by a further reduction in CDH1, and a large increase in ZEB1 expressions levels (Fig. S4C). Control MCF10A ER:HRAS V12 cells formed single round acini. Knockdown of *ATG5* in control MCF10A ER:HRAS V12 cells showed no significant effect. Induction of oncogenic *RAS* for 24 h before visualization resulted in acini invading into the Matrigel with short protrusions. When oncogenic *RAS* was induced in *ATG5*-depleted MCF10A cells, almost all acini produced more and longer protrusions compared to 4-OHT-treated cells (Fig. S4D, E).

These data demonstrate that *ATG5* knockdown promotes cell migration and invasion in *RAS*-mutated cells *in vitro*.

### Autophagy inhibition promotes oncogenic *ras*-induced EMT via the SQSTM1-RELA pathway

One of the key proteins involved in and regulated by autophagy is SQSTM1, which is a critical new player in cancer [40,41]. It has been suggested that sustained SQSTM1 expression resulting from autophagic defects is sufficient to activate the NFKB/NF-κB pathway and promote tumorigenesis [42]. SQSTM1 has also been indicated to be required for efficient tumorigenesis by oncogenic *RAS* [10,43]. Importantly, RELA of the NFKB pathway is one of the key transcription factors that can directly bind the promoters of *SNAI1, SNAI2, TWIST1, ZEB1* and *ZEB2* and induce their expressions [18,44–46]. These reports suggest a potential role of SQSTM1 in mediating the biological effects induced by autophagy inhibition in *RAS*-mutated cells.

**Figure 4.**
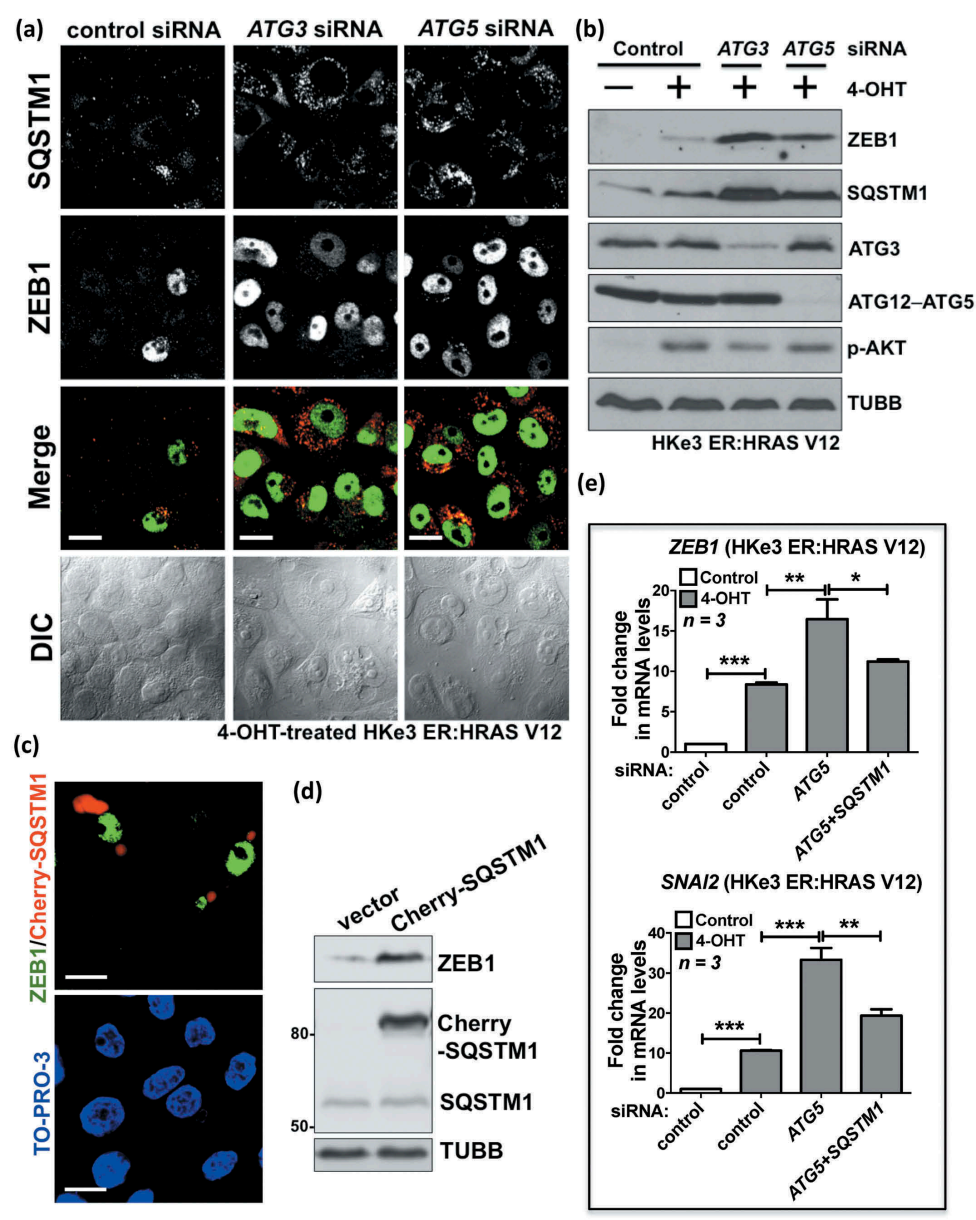
Autophagy inhibition promotes oncogenic *RAS*-induced EMT via SQSTM1. (a) Immunofluorescence staining of SQSTM1 (red) and ZEB1 (green), and differential interference contrast (DIC) images from 4-OHT-treated HKe3 ER:HRAS V12 cells with the indicated treatments. Scale bar: 20 μm. (b) Protein expression of ZEB1, SQSTM1, ATG3, ATG12–ATG5 and phospho-AKT (p-AKT) in HKe3 ER:HRAS V12 cells with the indicated treatments. TUBB was used as a loading control. (c) 4-OHT-treated HKe3 ER:HRAS V12 cells were transfected with Cherry-SQSTM1 (red) followed by immunofluorescence staining of ZEB1 (green). TO-PRO-3 (blue) was used to stain nucleic acids. Scale bar: 20 μm. (d) Protein expression of ZEB1, exogenously expressed Cherry-SQSTM1 and endogenous SQSTM1 in 4-OHT-treated HKe3 ER:HRAS V12 cells transfected with control vector or Cherry-SQSTM1. TUBB was used as a loading control. (**E**) Fold change in mRNA levels of *ZEB1* and *SNAI2* in HKe3 ER:HRAS V12 cells with the indicated treatments. mRNA levels normalized to *GAPDH* in control cells were used to set the baseline value at unity. Data are mean ± s.d. *n* = 3 samples per group. * *P* < 0.05. ** *P* < 0.01. *** *P* < 0.001.

In HKe3 ER:HRAS V12 cells, knockdown of *ATG3* or *ATG5* in the presence of 4-OHT resulted in SQSTM1 accumulation, concurrent with high levels of ZEB1 (Figure 4(a, b); Figure S5(a)). In addition, overexpression of exogenous SQSTM1 upregulated ZEB1 in 4-OHT-treated HKe3 ER:HRAS V12 cells (Figure 4(c)), confirmed by western blot analysis (Figure 4(d)). These data suggest that excess SQSTM1 upregulates ZEB1 levels. The role of SQSTM1 in autophagy inhibition-induced EMT in *RAS*-mutated cells was further confirmed by RNAi-mediated depletion of *SQSTM1*. As shown in Figure 4(e) and Figure S5(b), autophagy inhibition by knockdown of *ATG3* or *ATG5* further increased mutant *RAS*-induced expression of *ZEB1* or *SNAI2*, whereas depletion of *SQSTM1* partially abolished their induction, suggesting that SQSTM1 is at least one major mediator of this effect.

SQSTM1 is necessary for RAS to activate the NFKB pathway by triggering IKBKB/IκB kinase (part of the IKK complex) through the polyubiquitination of TRAF6 (TNF receptor associated factor 6) [43,47]. The NFKB pathway regulates ZEB1 or SNAI2 expression in mammary epithelial cells [45,46], and is essential for EMT and metastasis in a model of breast cancer progression [48]. This led us to hypothesize that SQSTM1 may induce EMT by activating the NFKB pathway.

**Figure 5.**
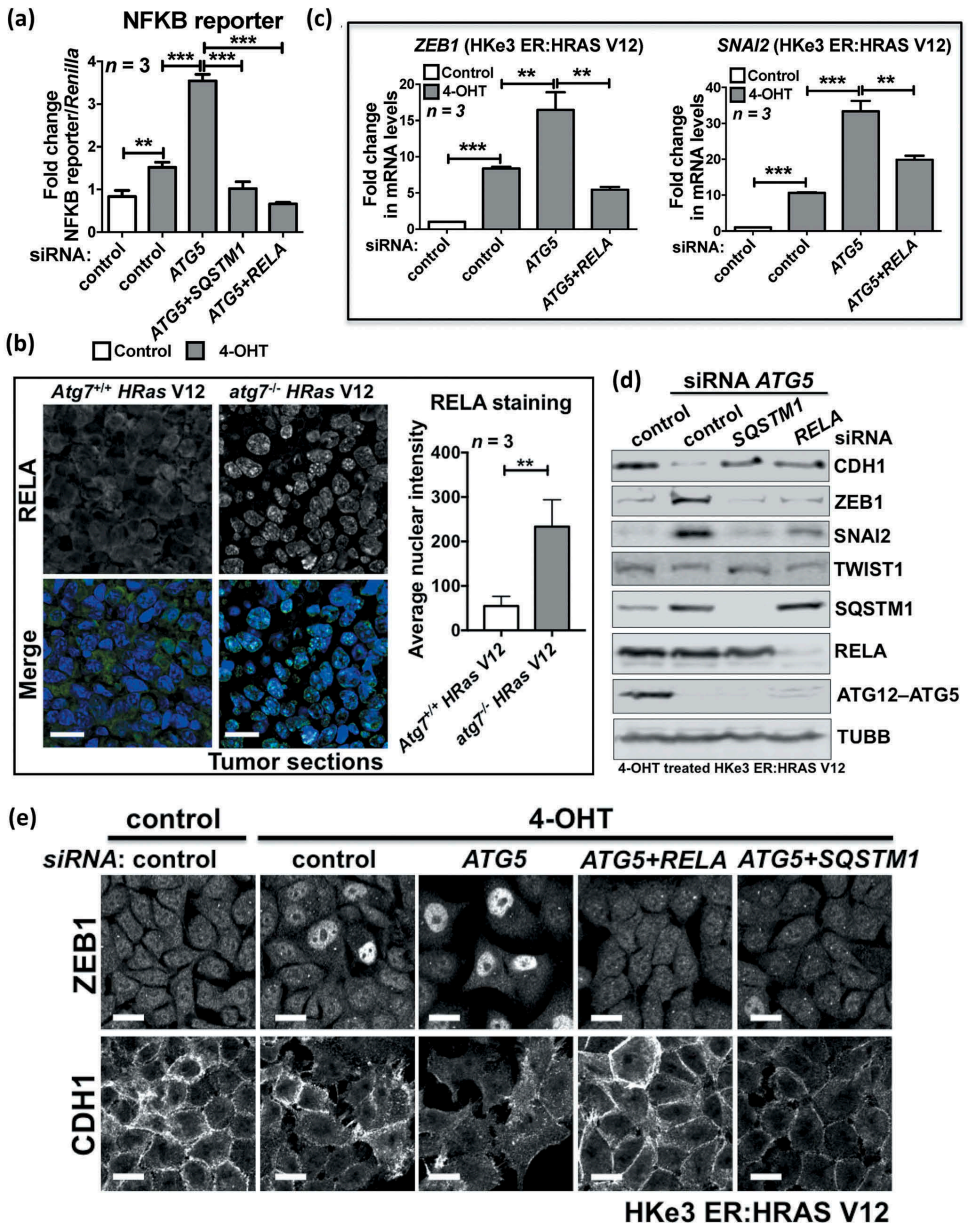
SQSTM1 accumulation resulting from autophagy inhibition in *RAS*-mutated cells activates the NFKB pathway to promote EMT. (a) Fold change in NFKB reporter activity in HKe3 ER:HRAS V12 cells with the indicated treatments. NFKB reporter luciferase readings normalized to *Renilla* in control cells were used to set the baseline value at unity. Data are mean ± s.d. *n* = 3 samples per group. ** *P* < 0.01. *** *P* < 0.001. (b) Immunofluorescence staining of RELA (green) in *HRas* V12-expressing *Atg7*^+/+^ or *HRas* V12-expressing *atg7*^−/-^ tumors. TO-PRO-3 (blue) was used to stain nucleic acids. Scale bar: 20 µm. *Atg7*^+/+^ or *atg7*^−/-^ iBMK cells transduced with *HRas* V12 were subcutaneously injected in nude mice to form tumors. The graph shows the average nuclear intensity of RELA evaluated with ImageJ, and data are mean ± s.d. *n* = 3 random fields. ** *P* < 0.01. (**C**) Fold change in mRNA levels of *ZEB1* and *SNAI2* in HKe3 ER:HRAS V12 cells with the indicated treatments. mRNA levels normalized to *GAPDH* in control cells were used to set the baseline value at unity. Data are mean ± s.d. *n* = 3 samples per group. ** *P* < 0.01. *** *P* < 0.001. (d) Protein expression of CDH1, ZEB1, SNAI2, TWIST1, SQSTM1, RELA and ATG12–ATG5 in HKe3 ER:HRAS V12 cells with the indicated treatments. TUBB was used as a loading control. (e) Immunofluorescence staining of ZEB1 or CDH1 in HKe3 ER:HRAS V12 cells with the indicated treatments. Scale bar: 20 μm.

To test this, we first assessed NFKB activity using a reporter assay under different conditions. Oncogenic *RAS* activation alone increased NFKB activity approximately 2-fold, while there was a nearly 6-fold increase in NFKB activity upon *ATG5* depletion (Figure 5(a)). Depletion of *SQSTM1* abolished this increase, comparable to the knockdown of *RELA* (Figure 5(a)), suggesting that autophagy inhibition in *RAS*-mutated cells triggers the NFKB pathway via SQSTM1. This point was further strengthened by the discovery that compared to the staining in oncogenic *RAS*-expressing *Atg5*^+/+^ or *Atg7*^+/+^ tumor cells, RELA was accumulated in the nuclei of oncogenic *RAS*-expressing *atg5*^−/-^ or *atg7*^−/-^ tumor cells *in vivo* (Figure 5(b); Figure S6(a)), indicating that the NFKB pathway is activated in *RAS*-mutated tumors when autophagy is inhibited.

Functionally, *RELA* knockdown abolished the increase in ZEB1 or SNAI2, and restored CDH1 expression following autophagy inhibition in *RAS*-mutated cells (Figure 5(c-e); Figure S6(b-e)). In HKe3 ER:HRAS V12 cells, *ATG3* or *ATG5* knockdown in the presence of 4-OHT dramatically increased *ZEB1* and *SNAI2* mRNA levels, whereas *RELA* depletion abolished this effect (Figure 5(c); Figure S6(c)), which was further confirmed by western blot analysis (Figure 5(d); Figure S6(b)) and immunofluorescence staining (Figure 5(e)). Depletion of *RELA* or *SQSTM1* was sufficient to restore the epithelial phenotype of HKe3 cells, demonstrated by the western blot (Figure 5(d)) and immunofluorescence staining of CDH1 (Figure 5(e)). Similar results were obtained from PANC1 cells (Fig. S6D, E). Knockdown of *ATG5* induced EMT in PANC1 cells, visible as decreased *CDH1* and increased *SNAI2* mRNA levels. The depletion of *SQSTM1* or *RELA* partially or completely abolished the increase in *SNAI2*, and restored *CDH1* expression.

**Figure 6.**
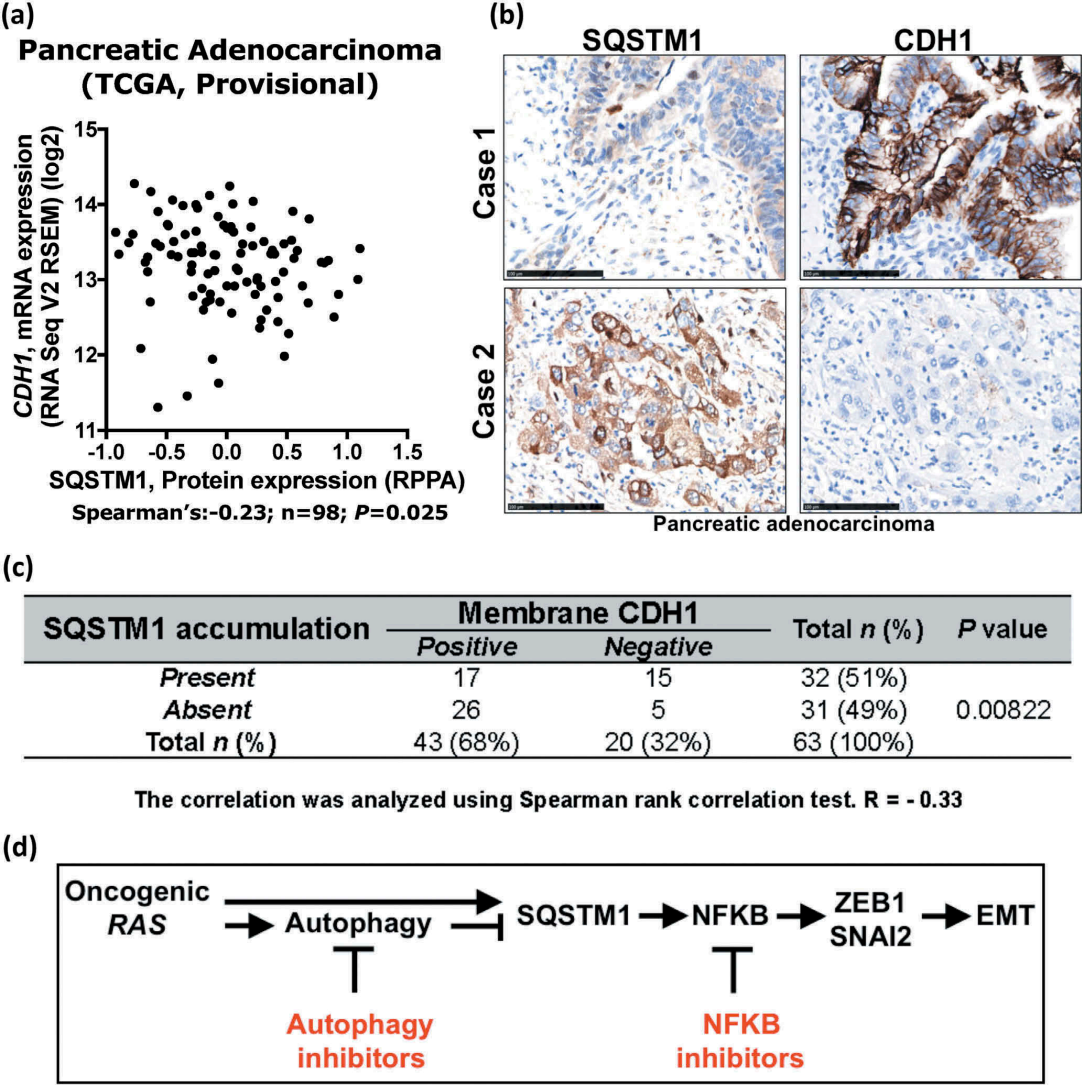
Accumulation of SQSTM1 correlates with negative membrane CDH1 expression in pancreatic adenocarcinoma. (a) The protein expression of SQSTM1 and mRNA level of *CDH1* is significantly inversely correlated in the TCGA pancreatic adenocarcinoma Provisional dataset (Spearman’s R = −0.23, *n* = 98, *P* = 0.025). (b) Adjacent tumor sections from representative cases show SQSTM1 and CDH1 expression in human pancreatic adenocarcinoma. Scale bar: 100 μm. (c) The relationship between SQSTM1 expression and protein expression of CDH1 was analyzed by Spearman rank correlation test (R = −0.33, *n* = 63, *P* = 0.00822). (d) Diagram summarizing the role of autophagy inhibition in oncogenic *RAS*-induced EMT (details are provided in the *Discussion*).

Finally, we assessed the expression of SQSTM1 and CDH1 in human pancreatic adenocarcinoma where 90% of tumors harbor *KRAS* mutations. In TCGA pancreatic adenocarcinoma Provisional dataset, we found that the SQSTM1 protein level was not significantly correlated with its mRNA level (Fig. S7A), which indicates a role for post-transcriptional regulation of SQSTM1 in pancreatic adenocarcinoma, consistent with the point that the SQSTM1 protein level is mainly regulated by the autophagic activity [40]. Using this dataset, we also found that the levels of SQSTM1 protein, but not mRNA, are significantly inversely correlated with *CDH1* mRNA level in this dataset (Figure 6(a), Pearson R = −0.22; n = 98; *P* = 0.03; Fig. S7B). Accumulation of SQSTM1 (Fig. S7C) correlated with loss of CDH1 expression (n = 63; *P* = 0.00822) in pancreatic adenocarcinoma (Figure 6(b, c)). These observations agree with the finding that SQSTM1 accumulation induces EMT. Taken together, these results demonstrate that autophagy inhibition in *RAS*-mutated cells promotes EMT via an SQSTM1-NFKB pathway.

## Discussion

Autophagy inhibition is a novel anticancer therapeutic strategy, with approximately 20 ongoing clinical trials employing this approach, either as monotherapy or in combination with other agents, in a variety of different cancers (http://www.cancer.gov/clinicaltrials). The initial interest in autophagy inhibition as an anticancer therapy was generated by research revealing that some cancers depend on autophagy for survival during external stresses such as hypoxia, chemotherapy or radiotherapy [15,49]. A new rationale for targeting autophagy has recently been elucidated by several groups, who have shown that elevated levels of autophagy are required for cancer cells to evade lethal metabolic stresses and maintain metabolic homeostasis, particularly in mutant *RAS*-driven tumorigenesis [6,8–10,12]. We have previously shown that autophagic activity helps bypass oncogenic *RAS*-induced senescence to allow transformation [11]. It was demonstrated recently that autophagy is a determinant of carcinoma fate, and that defects in the autophagic machinery may be a molecular basis for the occurrence of oncocytomas. *Atg7* was deleted concurrently with *KRas* G12D activation in mouse models for non-small cell lung cancer. *Atg7*-deficient tumors accumulate dysfunctional mitochondria and prematurely induce TRP53/p53 and proliferative arrest, which reduces tumor burden. This is partly relieved by *Trp53* deletion. Most interestingly, inflammatory responses are highly enriched and are responsible for the death of mice with *Atg7*-deficient tumors [7].

However, oncogenic *RAS* can trigger senescence and cell death via several essential autophagy proteins [50,51], consistent with prior reports that autophagy acts as a tumor suppressive mechanism [49,52,53]. Autophagy inhibition also significantly accelerates tumor formation in mice containing oncogenic *KRas*, but lacking *Trp53* [37]. Here, we report another reason for caution: autophagy inhibition in *RAS*-mutated cells induces EMT, which is associated with tumor invasion.

To inhibit autophagic activity, we depleted *ATG3* or *ATG5* with RNAi. We observed that autophagy inhibition in *RAS*-mutated cells, but not in wild-type *RAS*-expressing cells, significantly reduced CDH1 expression, and dramatically upregulated the expression of several transcriptional repressors of *CDH1*, in addition to promoting cell migration and invasion. Guo *et al*. reported previously that *Atg7* deficiency in tumor-derived cell lines causes more stromal infiltration in allograft tumors [7], while stromal infiltration is a predictor of tumor invasion in several cancer types [54–56]. These results together suggest that autophagy inhibition may promote tumor invasion *in vitro* and *in vivo*. Although EMT confers invasive features that allow cells to disseminate, the reverse process – mesenchymal-epithelial transition (MET) – is thought to allow the outgrowth of these cells at distant sites [37,57–59]. As a result, autophagy inhibition is not necessarily linked with enhanced metastasis and requires further investigation. To test the true impact of autophagy activity on tumor metastasis in vivo, a syngenic mouse model with switchable *Atg3* or *Atg5* in the presence or absence of oncogenic *RAS* is required, because we showed previously that autophagic activity dictates cellular response to oncogenic *RAS*-induced senescence [11], while senescent cells are able to secrete cytokines that will alter the tumor microenvironment, which is likely to have an impact on the metastatic potential in vivo [60]. Regardless, a number of other existing studies provide supporting evidence for a potential role of autophagy in inhibiting metastasis [29,34]. Rosenfeldt *et al*. demonstrated that the autophagy inhibitor hydroxychloroquine significantly accelerates tumor formation in mice containing oncogenic *KRas* but lacking *Trp53* [34]. Qin *et al*. showed that inhibition of autophagy in gastric cancer cells promotes EMT and liver metastasis [29].

We went on to confirm that this is achieved by the triggering of the NFKB pathway through the accumulation of SQSTM1. Coincidentally, Qiang and colleagues suggested that autophagy deficiency induces EMT via SQSTM1 through stabilization of TWIST1 [27]. SQSTM1 is required for efficient tumorigenesis by oncogenic *RAS* [10,43]. SQSTM1 is induced by mutant *RAS* through a mechanism that involves MAPK/ERK and PI3K, 2 bona fide downstream targets of RAS, and the AP1 element in the *SQSTM1* promoter [43]. Oncogenic *RAS* requires SQSTM1 to activate the NFKB pathway [43] for tumorigenesis. In addition, the identification of SQSTM1 as a new transcriptional target of NFKB is of particular importance [61]. Therefore, oncogenic *RAS* activates the expression of SQSTM1, either directly or via NFKB, to initiate a feed-forward loop to induce and sustain NFKB activity. Here, we show that triggering of the NFKB pathway by SQSTM1 is involved in the EMT induced by autophagy inhibition in *RAS*-mutated cells. Our data suggest that RAS signalling may induce SQSTM1 expression [43,61], while also activating autophagy to reduce its levels. However, inhibition of autophagy in the presence of a strong RAS signal causes an accumulation of SQSTM1, resulting in superactivation of the NFKB pathway, which leads to EMT (Figure 6(d)). Although it has been shown that activated RAS requires autophagy for tumorigenesis [8,10], oncogenic *RAS*-expressing autophagy-deficient cells can still form tumors in nude mice. Furthermore, we observed that the NFKB pathway is activated while CDH1 expression is reduced in these tumors, indicating that EMT is induced. As a result, autophagy inhibition in *RAS*-mutated cancers may help cancer cells detach from their neighboring cells and invade new sites. We also showed that depletion of *RELA*, a key component of the NFKB pathway, blocks autophagy inhibition-induced EMT in *RAS*-mutated cells. NFKB signalling is required for oncogenic *RAS*-induced lung tumorigenesis [62,63], and NFKB inhibitors have therapeutic efficacy in the treatment of lung cancer [64]. Our report suggests that a combination of autophagy inhibitors with NFKB inhibitors may therefore be necessary to treat *RAS*-mutated cancer (Figure 6(d)).

## Materials and methods

### Cell culture, reagents and transfections

MDA Panc3, PaCa3, Suit-2, PANC1, HKe3 ER:HRAS V12, HKh2, HKe3, HCT116 and HaCat cells were cultured in DMEM (Fisher Scientific UK, 11594446) supplemented with 10% FBS (Invitrogen, 10270106) and antibiotics. MCF10A ER:HRAS V12 cells were maintained in a 1:1 mixture of DMEM and Ham’s F12 medium (Fisher Scientific UK, 11524436) supplemented with 5% horse serum (Gibco, 26050088), 20 ng/ml EGF (Bio-techne, 236-EG), 100 ng/ml cholera toxin (Sigma, C8052), 10 μg/ml insulin (Sigma, I1882), 500 ng/ml hydrocortisone (Sigma, H0888) and antibiotics. All cells were kept at 37°C and 10% CO_2_. For 3D acini cultures, MCF10A ER:HRAS V12 cells were cultured as previously described [37] on growth factor-reduced Matrigel (BD Biosciences, 354230). Corning Cell Recovery Solution was used to recover cells from Matrigel. TGFB and 4-Hydroxytamoxifen (4-OHT) was purchased from PeproTech (100–21) and Sigma-Aldrich (H6278).

The cherry-SQSTM1 construct was obtained from Prof. Terje Johansen (University of Tromsø). Transfections were performed with FuGENE 6 (Promega, E2691), according to the manufacturer’s instructions.

siRNA oligos against *ATG5, ATG3, RELA* and *SQSTM1* were purchased from Dharmacon. Sequences are available from Dharmacon or upon request. As a negative control, we used siGENOME RISC-Free siRNA (Dharmacon, D-001220–01). MCF10A ER:HRAS V12 cells were transfected with the indicated siRNA oligos at a final concentration of 37.5 nM using Lullaby (OZ Biosciences, LL71000), according to the manufacturer’s instructions. Other cells were transfected with the indicated siRNA oligos at a final concentration of 35 nM using Dharmafect 1 reagent (Dharmacon, T-2001–03).

Stable knockdown of human *ATG5* was carried out using pLVX-shRNA-mCherry-Hygro lentiviral expression plasmid. The *ATG5* shRNA plasmid was designed to target the sequence 5ʹ-CCTGAACAGAATCATCCTTAA-3ʹ.

### Western blot analysis

Western blot analysis was performed with lysates from cells or tissues with urea buffer (8 M urea [Sigma, U5378], 1 M thiourea [Sigma, T8656], 0.5% CHAPS [Fisher Scientific, 11461851], 50 mM DTT [Fisher Scientific UK, 10375600], 24 mM spermine [Sigma, 85,590]). Primary antibodies were from: Santa Cruz Biotechnology (ACTB/β-actin, sc-47778; ZEB1, sc-25388; ZEB2, sc-48789; CDH1, sc-21791; SNAI2, sc-10436), Abcam (TUBB/β1-tubulin, ab6046; GAPDH, ab9385), Cell Signaling Technology (ATG5, 2630; ATG3, 3415; LC3, 3868; phospho-AKT, 9271; AKT, 4685; phospho-MAPK, 4370; SNAI1, 3879; SNAI2, 9585; RELA, 8242; TWIST1, 46702; phospho-Smad2, 3104; Smad2, 5339), Progen Biotechnik GmbH (SQSTM1, GP62-C), BD Transduction Laboratories (CDH1, 610405; SQSTM1, 610833). Signals were detected using an ECL detection system (GE Healthcare, RPN2108) or Odyssey imaging system (LI-COR, United States), and evaluated by ImageJ 1.42q software (National Institutes of Health, United States).

### Quantitative reverse transcription-PCR

The real-time RT-PCR was performed using gene-specific primers (QuantiTect Primer Assays, Qiagen) for *CDH1, VIM, ZEB1*/*2, SNAI1*/*2, TWIST1*, or *GAPDH* with QuantiTect SYBR Green RT-PCR Kits (Qiagen, 204243). Relative transcript levels of target genes were normalized to *GAPDH* mRNA levels.

### Luciferase reporter assay

HKe3 ER:HRAS V12 cells were transfected with the indicated siRNAs for 48 h in 24-well plates, followed by 24-h transfection with 250 ng of NFKB reporter and 10 ng of phRL-CMV (Promega, E2261), which constitutively expresses the *Renilla* luciferase reporter. One day before the measurement of luciferase activity 100 nM 4-OHT was added. Finally, the transcription assay was carried out using the Dual-luciferase® reporter assay system (Promega, E1960) following the manufacturer’s protocol.

### Wound-healing migration, transwell migration and matrigel invasion assays

The wound-healing migration assays were done in conjunction with siRNA transfections in control or 4-OHT-treated HKe3 ER:HRAS V12 cells. Seventy-two h after siRNA transfections, confluent monolayers of cells were wounded with a p20 pipette tip (time 0). Phase-contrast images were taken using an Olympus inverted microscope at time 0 h or 24 h after the scratch wound. For the Transwell migration assay, Transwell membranes (8-μm pore size, 6.5-mm diameter; Corning Costar, 3422) were used. The bottom chambers of the Transwell were filled with migration-inducing medium (with 50% FBS). The top chambers were seeded with 1.5 × 10^5^ live serum-starved control or *ATG5* shRNA HCT116 cells per well. After 48 h, the filters were fixed with 4% paraformaldehyde for 10 min at room temperature; subsequently, the cells on the upper side of the membrane were scraped with a cotton swab. Similar inserts coated with Matrigel (Corning, 354480) were used to determine invasive potential in invasion assays. Filters were stained with crystal violet for light microscopy. Images were taken using an Olympus inverted microscope and migratory cells were evaluated by ImageJ 1.42q software (National Institutes of Health, United States).

### Immunofluorescence

Cells were fixed in 4% PBS (Fisher Scientific UK, 12579099)-paraformaldehyde for 15 min, incubated in 0.1% Triton-X-100 (Fisher Scientific UK, 11471632) for 5 min on ice, then in 0.2% fish skin gelatin (Sigma, G7041) in PBS for 1 h and stained for 1 h with an anti-CDH1 (Santa Cruz Biotechnology, sc-21791; 1:100), anti-ZEB1 (Santa Cruz Biotechnology, sc-25388; 1:100), anti-LC3 (Cell Signaling Technology, 3868; 1:100) or anti-SQSTM1 (Progen Biotechnik GmbH, GP62-C; 1:300) antibody. Protein expression was detected using Alexa Fluor 488 or 546 (1:400; Fisher Scientific UK, 10256302,10789154,10729174,10002502,10717474) for 20 min. TO-PRO-3 (Invitrogen, T3605: 1:1000) was used to stain nucleic acids. Rhodamine-phalloidin (Molecular Probes, R415) was used to visualize filamentous actin (F-actin). For immunofluorescence staining of 3D cultures from MCF10A ER:HRAS V12 cells, acini were fixed with 4% paraformaldehyde for 40 min, permeabilized in 0.5% Triton X-100 for 10 min on ice and stained with Rhodamine-phalloidin for 1 h at room temperature. Acini were counterstained with DAPI. For tissue immunofluorescence staining, rehydrated paraffin-embedded sections were microwaved in 10 mM sodium citrate buffer (pH 6) to unmask the antigen, washed with PBS, and incubated overnight with the primary antibody at 4°C, followed by Alexa Fluor 488 or 546 (1:400; Fisher Scientific UK, 10256302,10789154,10729174,10002502) secondary antibody for 60 min at room temperature. The sections were incubated with primary antibodies against CDH1 (Santa Cruz Biotechnology, sc-21791; 1:200), or RELA (Santa Cruz Biotechnology, sc-372; 1:200). When grown in nude mice, nontumorigenic baby mouse kidney epithelial (iBMK) cells transduced with *HRas* V12 formed tumors [10]. Oncogenic *Ras* (*HRas* V12)-expressing *atg5*^−/-^ or *atg7*^−/-^ iBMK tumor sections and oncogenic *Ras* (*HRas* V12)-expressing *Atg5*^+/+^ or *Atg7*^+/+^ iBMK tumor sections were obtained from Prof. Eileen White (Rutgers Cancer Institute of New Jersey), described in an earlier publication in detail [10]. Samples were observed using a confocal microscope system (LSM 510 or LSM 710; Carl Zeiss, Germany). Acquired images were analyzed using Photoshop (Adobe Systems, United States) according to the guidelines of the journal.

### Immunohistochemical analysis

A pancreatic tissue microarray (reference no. HPan-Ade150CS-01), consisting of pancreatic adenocarcinoma and matched normal adjacent pancreatic tissue, was obtained from Shanghai Outdo Biotech Co., Ltd. (Shanghai, China). Sections were de-waxed, rehydrated and incubated with 3% hydrogen peroxide in methanol to block endogenous peroxidase activity (10 min). Sections were then blocked with normal goat serum (Fisher Scientific UK, 11819220) and incubated overnight at 4°C with a primary antibody against CDH1 (1:250: Cell Signaling Technology, 14472) or SQSTM1 (1:200: Cell Signaling Technology, 88588), followed by the biotinylated secondary antibody (Vector Labs, BA-1400, BA-1000; 1:250) for 40 min at room temperature. The staining reaction was worked up using the Vector Elite ABC kit (Vector Labs, PK-7200) and counterstained with haematoxylin. The staining intensity was graded as follows: No staining (0), weak (1), moderate (2) and intense staining (3). Samples that could not be graded were scored as ‘not applicable’. For each sample, membrane expression of CDH1 was determined as the average score of the sample spot, and then further subgrouped into negative (score 0) or positive (scores 1–3); while SQSTM1 accumulation present or absent was determined by comparing the pancreatic adenocarcinoma to the matched normal adjacent pancreatic tissue. Statistical analysis was undertaken in SPSS. The relationship between SQSTM1 accumulation and membrane expression of CDH1 was analyzed using the Spearman rank correlation test.

### TCGA analysis

Information on *CDH1* mRNA expression and SQSTM1 protein expression was examined using The Cancer Genome Atlas (TCGA) http://www.cbioportal.org/index.do [65,66]. Statistical analysis was undertaken in SPSS. The relationship between SQSTM1 protein expression and *SQSTM1* or *CDH1* mRNA expression was analyzed using the Spearman rank correlation test.

### Statistical analysis and repeatability of experiments

Each experiment was repeated at least twice. Unless otherwise noted, data are presented as mean ± s.d., and a two-tailed, unpaired Student’s *t*-test was used to compare 2 groups for independent samples. *P* < 0.05 was considered statistically significant.
