# Supplementary material for: Autophagy inhibition specifically promotes epithelial-mesenchymal transition and invasion in RAS-mutated cancer cells
Source: Autophagy. 2019 Feb 20;15(5):886–99. doi: 10.1080/15548627.2019.1569912 (PMC6517269; doi:10.1080/15548627.2019.1569912)
Supplement: Supplemental Material [file kaup-15-05-1569912-s001.zip › 1569912_supplementary information/downloadFromZipFile.pdf]

Wang_FigS1

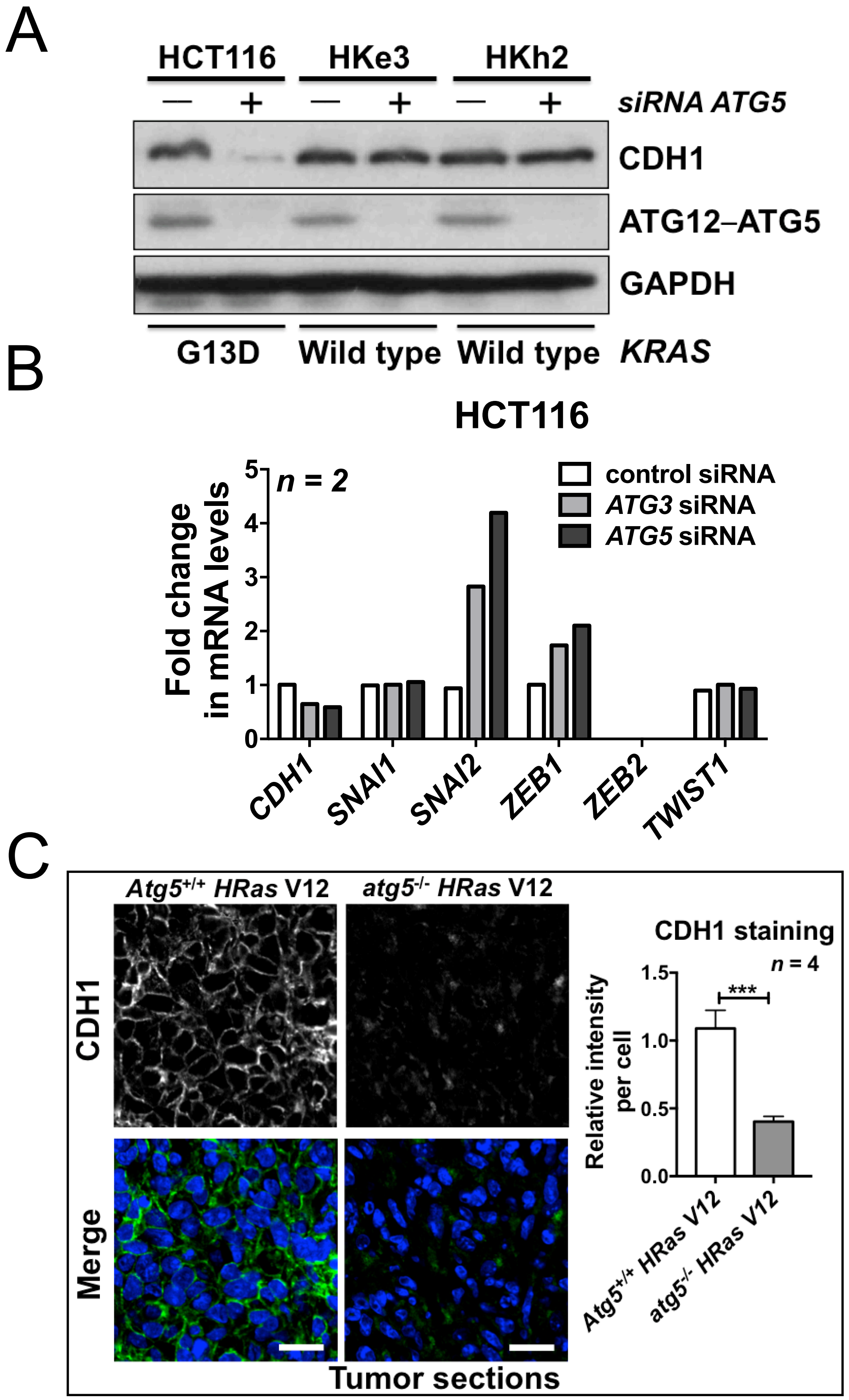
A
HCT116
HKe3
HKh2
—
+
—
+
—
+
siRNA ATG5
CDH1
ATG12–ATG5
GAPDH
G13D
Wild type
Wild type
KRAS
B
HCT116
Fold change in mRNA levels
n = 2
control siRNA
ATG3 siRNA
ATG5 siRNA
5
4
3
2
1
0
CDH1
SNAI1
SNAI2
ZEB1
ZEB2
TWIST1
C
Atg5+/+ HRas V12
atg5-/- HRas V12
CDH1
Merge
Tumor sections
CDH1 staining
n = 4
***
Relative intensity per cell
1.5
1.0
0.5
0.0
Atg5+/+ HRas V12
atg5-/- HRas V12

Wang_FigS2

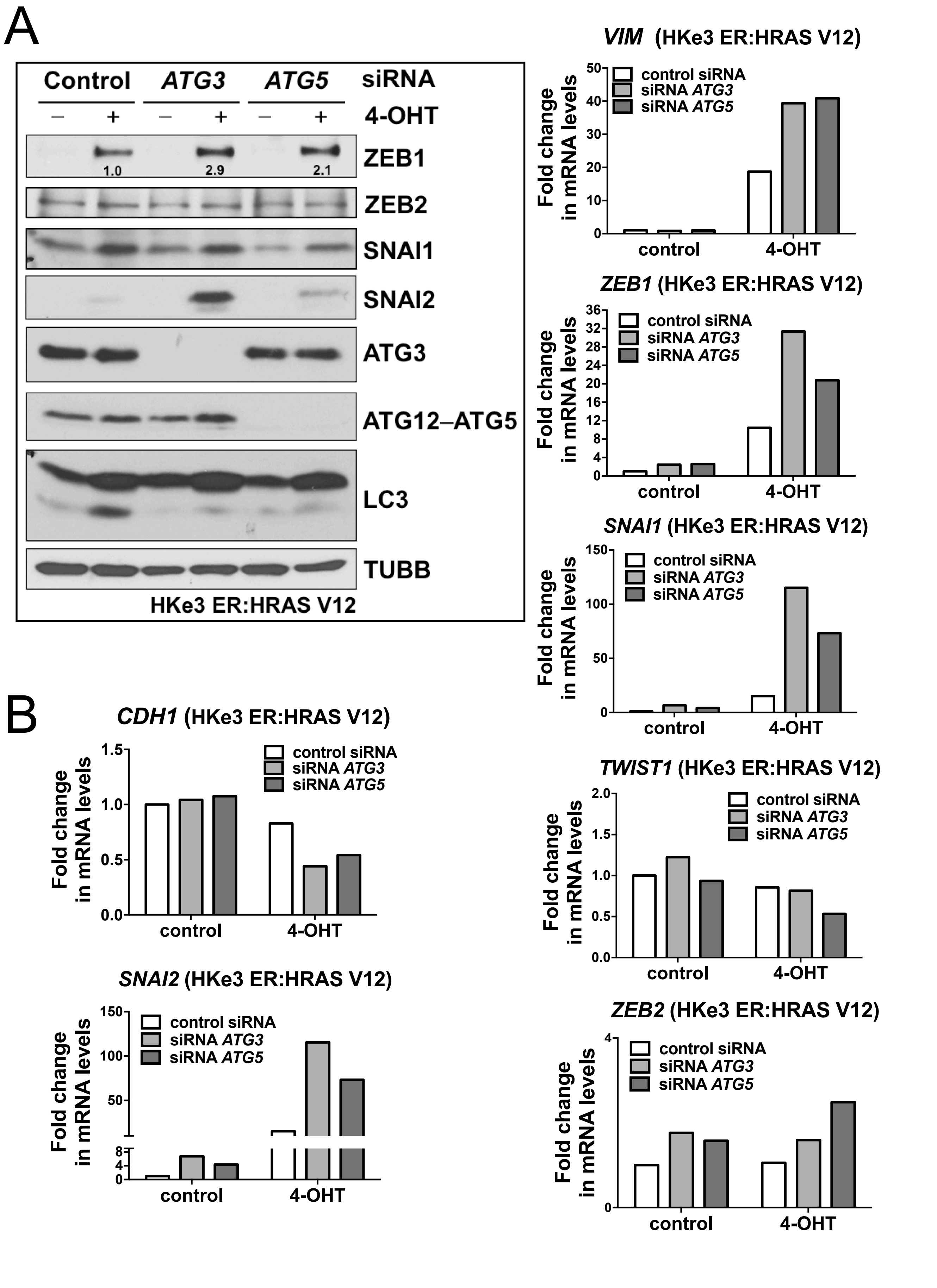
A
Control
ATG3
ATG5
siRNA
4-OHT
ZEB1
1.0
2.9
2.1
ZEB2
SNAI1
SNAI2
ATG3
ATG12–ATG5
LC3
TUBB
HKe3 ER:HRAS V12
VIM (HKe3 ER:HRAS V12)
ZEB1 (HKe3 ER:HRAS V12)
SNAI1 (HKe3 ER:HRAS V12)
TWIST1 (HKe3 ER:HRAS V12)
ZEB2 (HKe3 ER:HRAS V12)
B
CDH1 (HKe3 ER:HRAS V12)
SNAI2 (HKe3 ER:HRAS V12)
Fold change in mRNA levels
control siRNA
siRNA ATG3
siRNA ATG5
control
4-OHT

Wang_FigS3

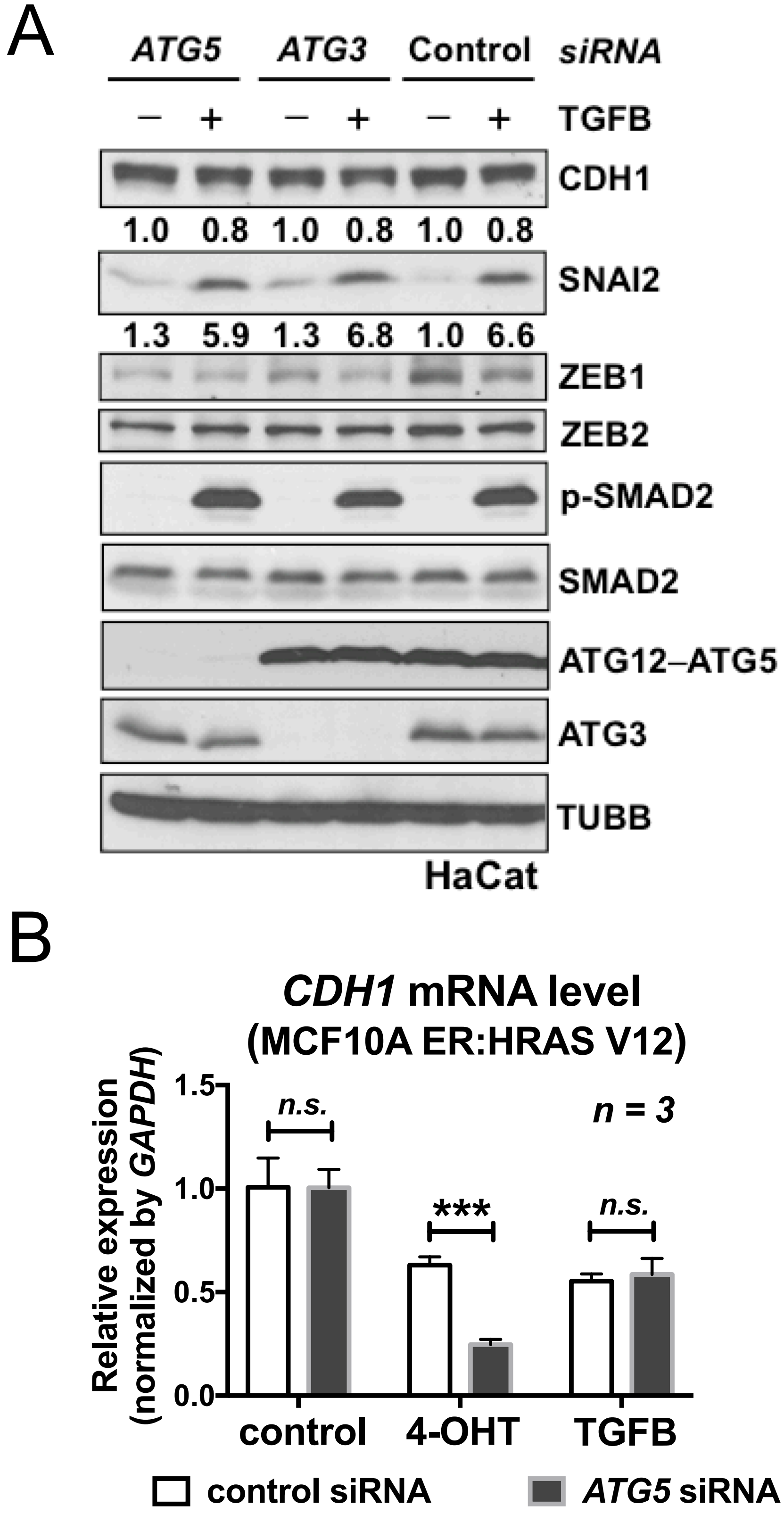
A
ATG5
ATG3
Control
siRNA
– + – + – +
TGFB
CDH1
1.0 0.8 1.0 0.8 1.0 0.8
SNAI2
1.3 5.9 1.3 6.8 1.0 6.6
ZEB1
ZEB2
p-SMAD2
SMAD2
ATG12–ATG5
ATG3
TUBB
HaCat
B
CDH1 mRNA level
(MCF10A ER:HRAS V12)
Relative expression
(normalized by GAPDH)
1.5
1.0
0.5
0.0
n.s.
***
n.s.
n = 3
control
4-OHT
TGFB
control siRNA
ATG5 siRNA

Wang_FigS4

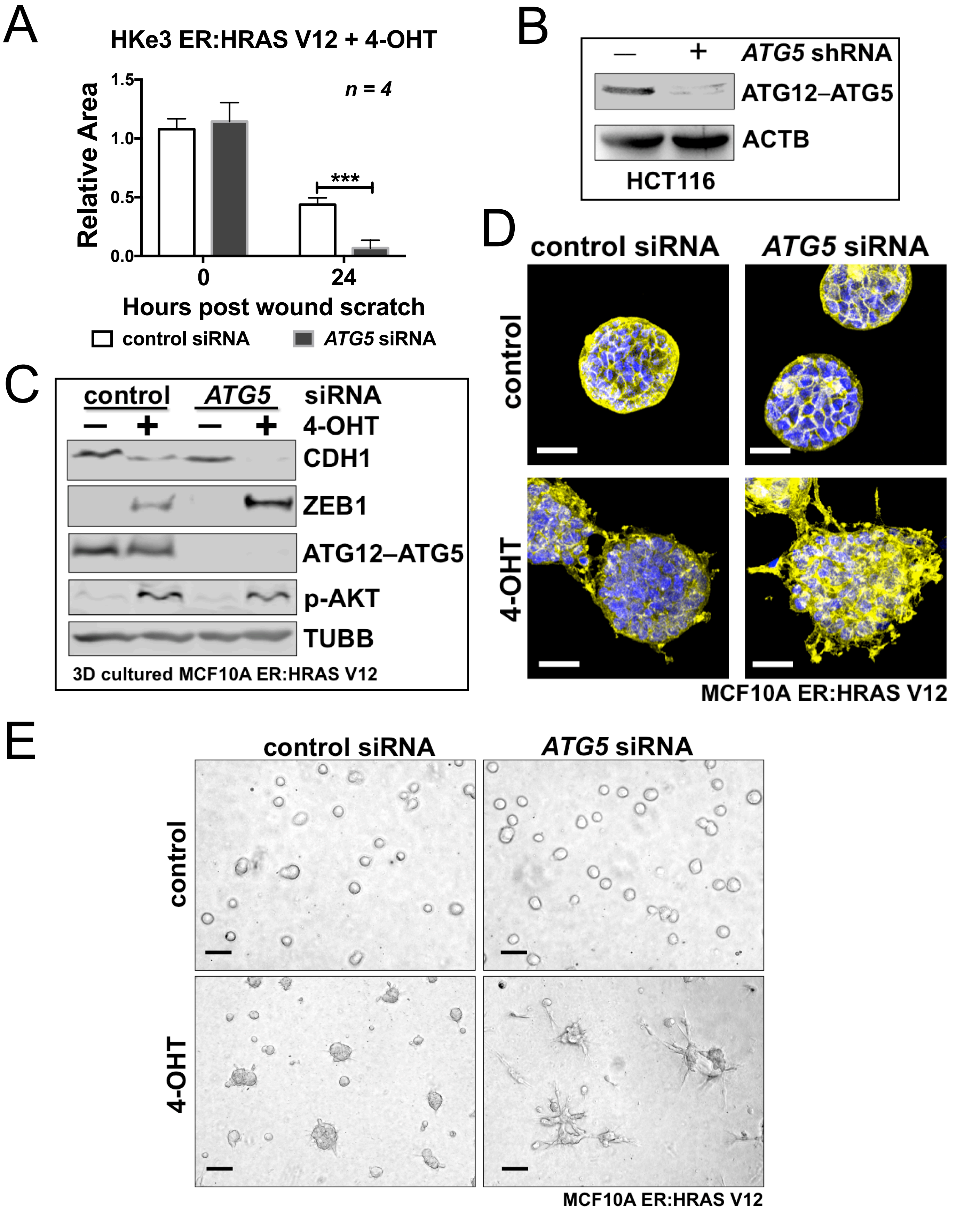
A
HKe3 ER:HRAS V12 + 4-OHT
n = 4
Relative Area
1.5
1.0
0.5
0.0
***
0
24
Hours post wound scratch
control siRNA
ATG5 siRNA
B
— + ATG5 shRNA
ATG12–ATG5
ACTB
HCT116
C
control ATG5 siRNA
— + — + 4-OHT
CDH1
ZEB1
ATG12–ATG5
p-AKT
TUBB
3D cultured MCF10A ER:HRAS V12
D
control siRNA
ATG5 siRNA
control
4-OHT
MCF10A ER:HRAS V12
E
control siRNA
ATG5 siRNA
control
4-OHT
MCF10A ER:HRAS V12

Wang_FigS5

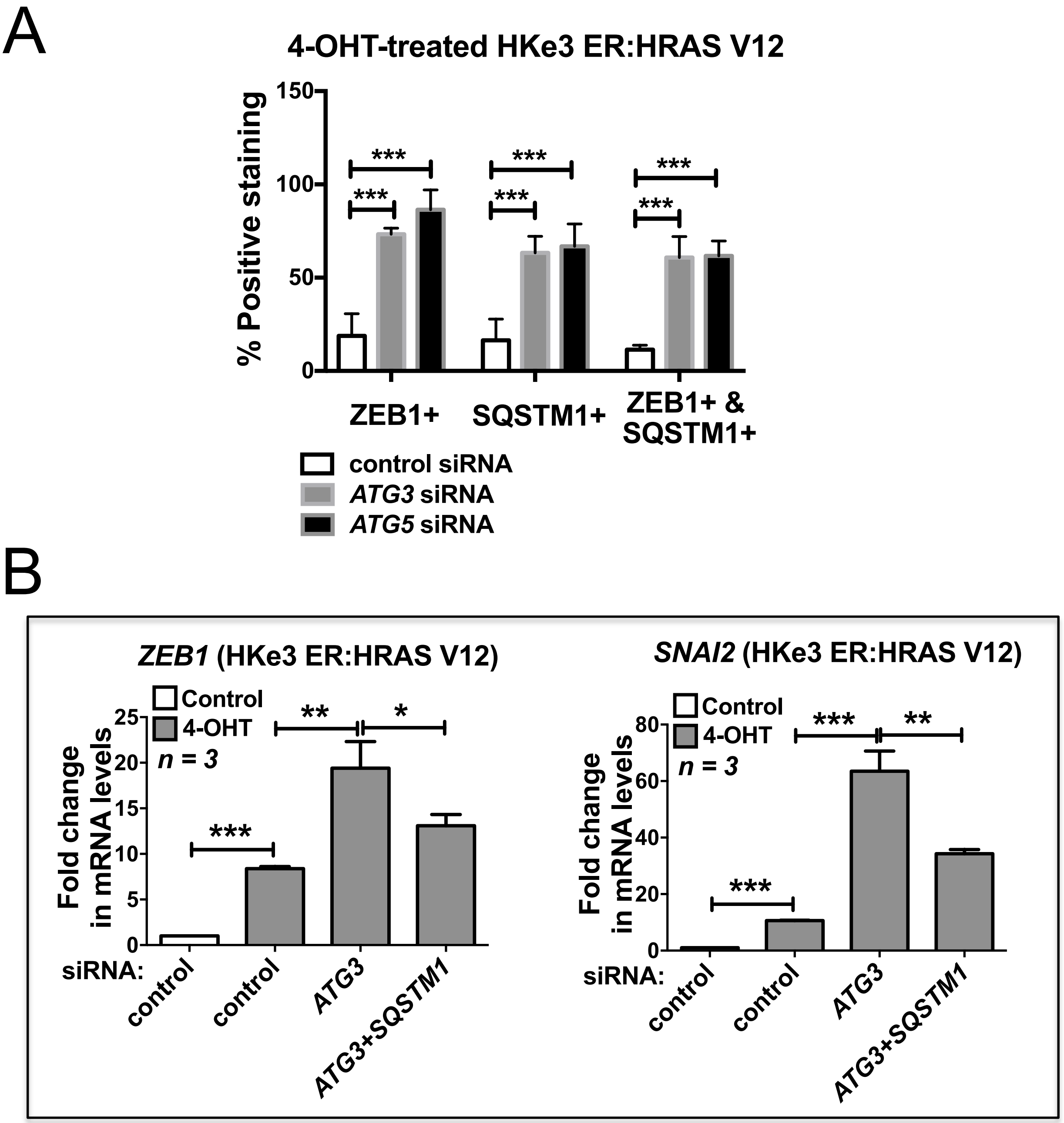
A
4-OHT-treated HKe3 ER:HRAS V12
% Positive staining
150
100
50
0
***
ZEB1+
SQSTM1+
ZEB1+ &
SQSTM1+
control siRNA
ATG3 siRNA
ATG5 siRNA
B
ZEB1 (HKe3 ER:HRAS V12)
Control
4-OHT
n = 3
Fold change
in mRNA levels
25
20
15
10
5
0
***
**
*
siRNA:
control
control
ATG3
ATG3+SQSTM1
SNAI2 (HKe3 ER:HRAS V12)
Control
4-OHT
n = 3
Fold change
in mRNA levels
80
60
40
20
0
***
***
**
siRNA:
control
control
ATG3
ATG3+SQSTM1

Wang_FigS6

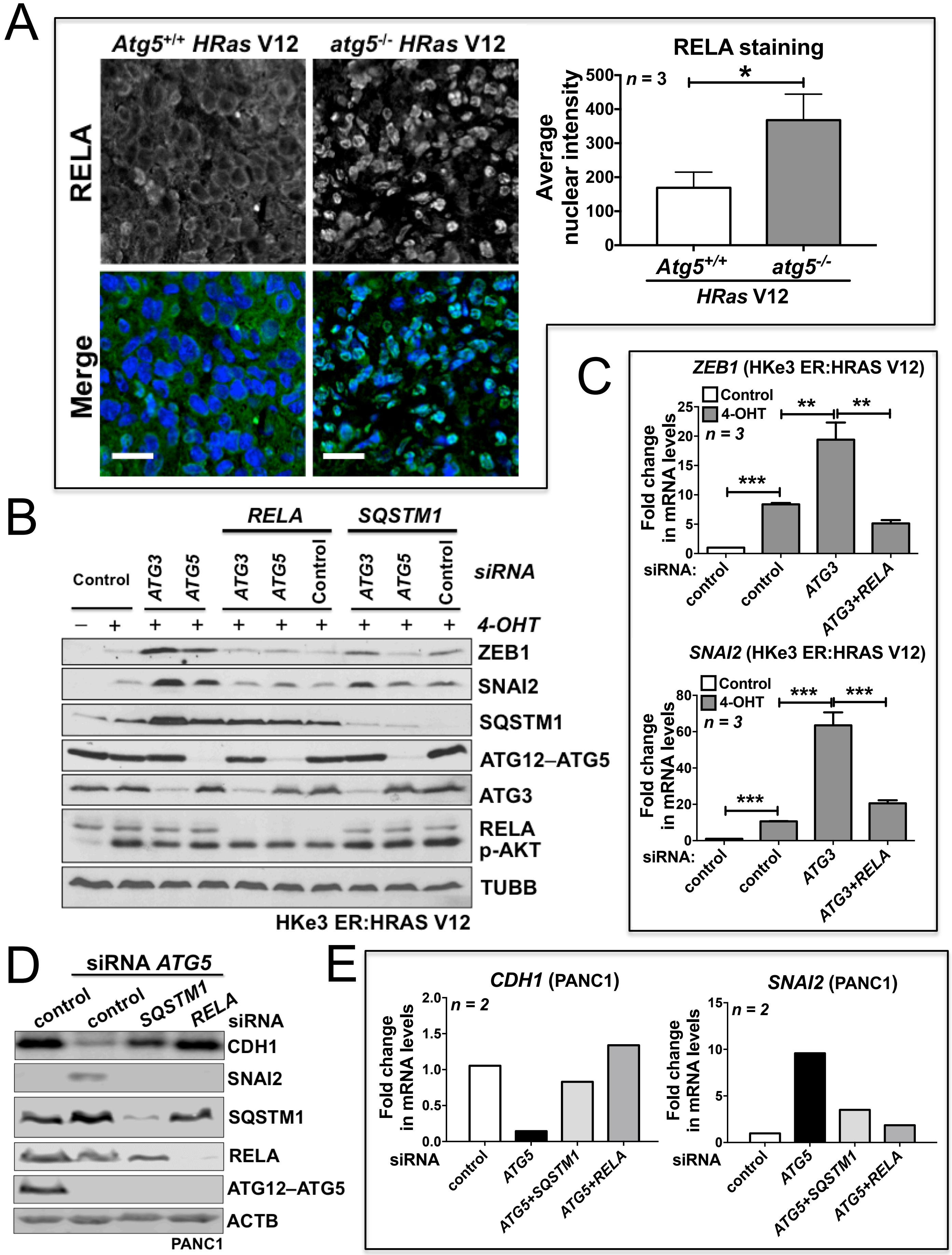
A
Atg5+/+ HRas V12
atg5-/- HRas V12
RELA
Merge
RELA staining
n = 3
Average nuclear intensity
Atg5+/+
atg5-/-
HRas V12
B
RELA
SQSTM1
Control
ATG3
ATG5
siRNA
4-OHT
ZEB1
SNAI2
SQSTM1
ATG12–ATG5
ATG3
RELA
p-AKT
TUBB
HKe3 ER:HRAS V12
C
ZEB1 (HKe3 ER:HRAS V12)
Control
4-OHT
n = 3
Fold change in mRNA levels
siRNA:
control
ATG3
ATG3+RELA
SNAI2 (HKe3 ER:HRAS V12)
D
siRNA ATG5
control
SQSTM1
RELA
siRNA
CDH1
SNAI2
SQSTM1
RELA
ATG12–ATG5
ACTB
PANC1
E
CDH1 (PANC1)
SNAI2 (PANC1)
n = 2
ATG5
ATG5+SQSTM1
ATG5+RELA

Wang_FigS7

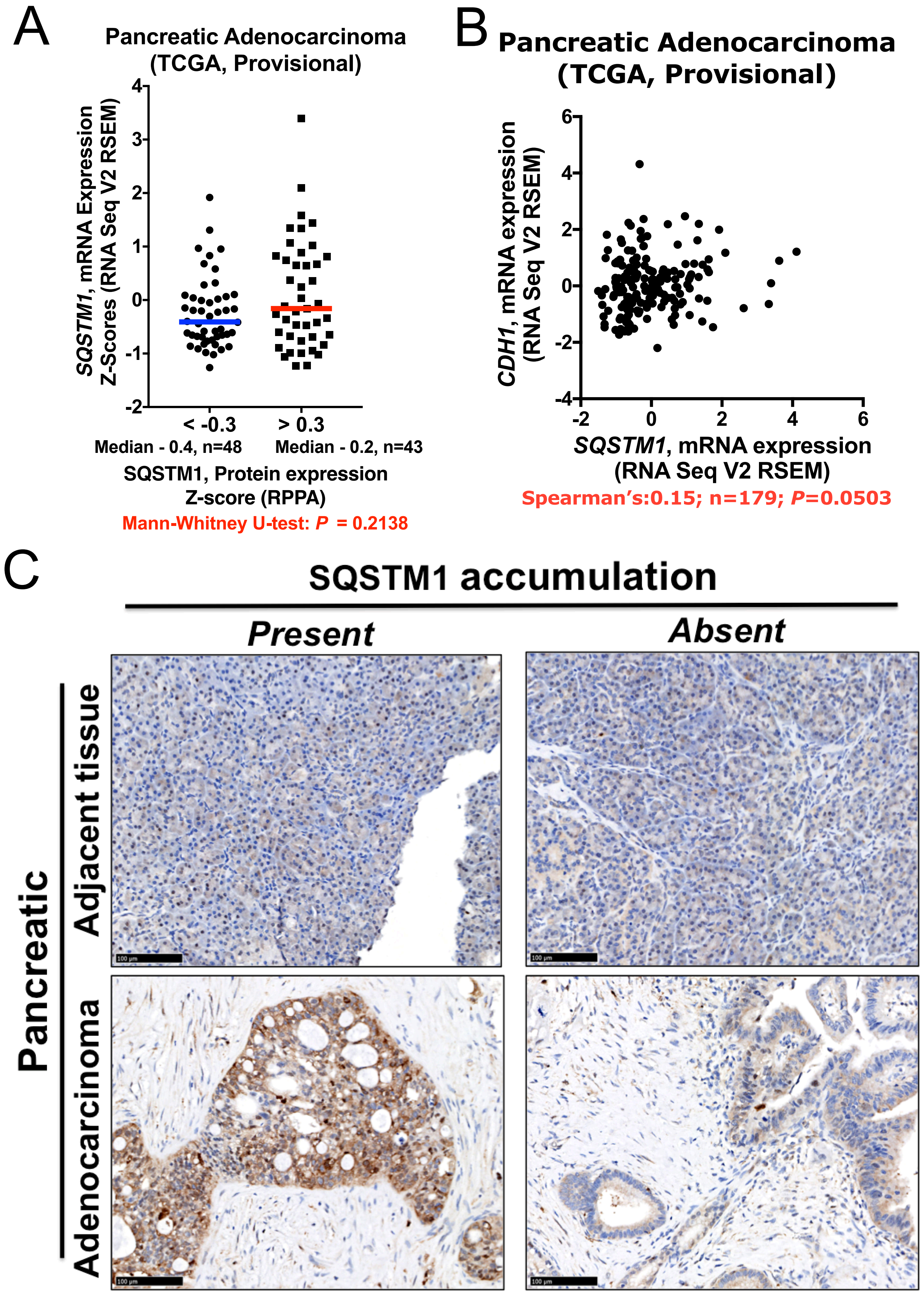

A
Pancreatic Adenocarcinoma
(TCGA, Provisional)
SQSTM1, mRNA Expression
Z-Scores (RNA Seq V2 RSEM)
4
3
2
1
0
-1
-2
< -0.3
> 0.3
Median - 0.4, n=48
Median - 0.2, n=43
SQSTM1, Protein expression
Z-score (RPPA)
Mann-Whitney U-test: P = 0.2138
B
Pancreatic Adenocarcinoma
(TCGA, Provisional)
CDH1, mRNA expression
(RNA Seq V2 RSEM)
6
4
2
0
-2
-4
-2
0
2
4
6
SQSTM1, mRNA expression
(RNA Seq V2 RSEM)
Spearman's:0.15; n=179; P=0.0503
C
SQSTM1 accumulation
Present
Absent
Pancreatic
Adjacent tissue
Adenocarcinoma
