# Supplementary material for: Autophagy inhibition specifically promotes epithelial-mesenchymal transition and invasion in RAS-mutated cancer cells
Source: Autophagy. 2019 Feb 20;15(5):886–99. doi: 10.1080/15548627.2019.1569912 (PMC6517269; doi:10.1080/15548627.2019.1569912)
Supplement: Supplemental Material [file kaup-15-05-1569912-s001.zip › 1569912_supplementary information/SI text-280918.doc]

**Supplementary Figure Legends**

**Figure S1.** Autophagy inhibition promotes EMT in *RAS*-mutated cells. (**A**) Protein expression of CDH1 and ATG12–ATG5 in colorectal cancer cell lines with the indicated transfections. GAPDH was used as a loading control. *KRAS* mutation status is also indicated. (**B**) Fold change in mRNA levels of *CDH1*, *SNAI1*, *SNAI2*, *ZEB1*, *ZEB2* and *TWIST1* in HCT116 cells with the indicated siRNA transfections. mRNA levels normalized to *GAPDH* in control cells were used to set the baseline value at unity. Data are the means of 2 independent experiments carried out in duplicate. (**C**) Immunofluorescence staining of CDH1 (green) in *HRas* V12-expressing *Atg5*^+/+^ tumors or *HRas* V12-expressing *atg5*^-/-^ tumors. TO-PRO-3 (blue) was used to stain nucleic acids. *Atg5*^+/+^ or *atg5*^-/-^ iBMK cells transduced with *HRas* V12 were subcutaneously injected in nude mice to form tumors. Scale bar: 20 μm*.* The graph shows the intensity of CDH1 evaluated with ImageJ, and data are mean ± s.d. *n* = 4 random fields. *** *P* < 0.001.

**Figure S2.** Autophagy inhibition promotes oncogenic *RAS*-induced EMT. (**A**) Protein expression of ZEB1, ZEB2, SNAI1, SNAI2, ATG3, ATG12–ATG5 and LC3 in HKe3 ER:HRAS V12 cells with the indicated treatments. TUBB was used as a loading control. The level of ZEB1 was calculated by densitometry and the fold increase calculated by normalizing it against control siRNA transfections in 4-OHT-treated cells (1.0). (**B**) Fold change in mRNA levels of *CDH1*, *VIM*, *SNAI2*, *ZEB1*, *SNAI1*, *TWIST1* and *ZEB2* in HKe3 ER:HRAS V12 cells with the indicated treatments. mRNA levels normalized to *GAPDH* in control siRNA transfected control cells were used to set the baseline value at unity. Data are the means of 2 independent experiments carried out in duplicate.

**Figure S3.** Autophagy inhibition does not promote TGFB-induced EMT. (**A**) Protein expression of CDH1, SNAI2, ZEB1, ZEB2, phospho-SMAD2 (p-SMAD2), SMAD2, ATG12–ATG5 and ATG3 in HaCat cells with the indicated treatment. TUBB was used as a loading control. The level of CDH1 or SNAI2 was calculated by densitometry and the fold increase calculated by normalizing it against control cells (1.0). (**B**) Fold change in mRNA levels of *CDH1* in MCF10A ER:HRAS V12 cells with the indicated treatments. mRNA levels normalized to *GAPDH* in control cells were used to set the baseline value at unity. Data are mean ± s.d. *n* = 3 samples per group. *n.s. P* > 0.05, *** *P* < 0.001.

**Figure S4.** Autophagy inhibition promotes oncogenic *RAS*-induced cell migration and invasion. (**A**) Scratch wound assay of 4-OHT-treated HKe3 ER:HRAS V12 cells transfected with control or *ATG5* RNAi. The graph shows the area of a wound evaluated with ImageJ, and data are mean ± s.d. *n* = 4. *** *P* < 0.001. (**B**) Western blot analysis of lysates from HCT116 cells infected with control or *ATG5* shRNA showing effects on ATG12–ATG5. ACTB was used as a loading control. (**C**) Western blot analysis of lysates from 3D-cultured MCF10A ER:HRAS V12 cells in Matrigel with the indicated treatments showing effects on CDH1, ZEB1, ATG12–ATG5, and phospho-AKT (p-AKT). TUBB was used as a loading control. (**D**) Representative 3D reconstruction confocal images of MCF10A ER:HRAS V12 cells cultured in Matrigel with the indicated treatments. MCF10A ER:HRAS V12 cells were transfected with control or *ATG5* siRNA for 5 days, followed by 100 nM 4-OHT treatment for 1 day. Acini were stained for F-actin with rhodamine-phalloidin (yellow) and DAPI (blue). Scale bar: 40 μm*.* (**E**) Representative phase contrast images of MCF10A-ER:HRASV12 cells cultured in Matrigel with the indicated treatments. Scale bar: 200 μm*.*

**Figure S5.** Autophagy inhibition promotes oncogenic *RAS*-induced EMT via SQSTM1. (**A**) Knockdown of ATG3 or ATG5 in RAS-activated cells results in SQSTM1 accumulation, concurrent with high levels of ZEB1. The graph shows the percentage of positive cells in ZEB1, SQSTM1 or both with the indicated transfections. Data are mean ± s.d. *n* = 4. *** *P* < 0.001. Images are shown in Fig. 4A. (**B**) Fold change in mRNA levels of *ZEB1* and *SNAI2* in HKe3 ER:HRAS V12 cells with the indicated treatments. mRNA levels normalized to *GAPDH* in control cells were used to set the baseline value at unity. Data are mean ± s.d. *n* = 3 samples per group. * *P* < 0.05. ** *P* < 0.01. *** *P* < 0.001.

**Figure S6.** SQSTM1 accumulation resulting from autophagy inhibition in *RAS*-mutated cells activates the NFKB pathway to promote EMT. (**A**) Immunofluorescence staining of RELA (green) in *HRas* V12-expressing *Atg5*^+/+^ or *HRas* V12-expressing *atg5*^-/-^ tumors. TO-PRO-3 (blue) was used to stain nucleic acids. Scale bar: 20 μm*. Atg5*^+/+^ or *atg5*^-/-^ iBMK cells transduced with *HRas* V12 were subcutaneously injected in nude mice to form tumors. The graph shows the average nuclear intensity of RELA evaluated by ImageJ, and data are mean ± s.d. *n* = 3 random fields. * *P* < 0.05. (**B**) Protein expression of ZEB1, SNAI2, SQSTM1, ATG12–ATG5, ATG3, RELA and phospho-AKT (p-AKT) in HKe3 ER:HRAS V12 cells with the indicated treatments. TUBB was used as a loading control. (**C**) Fold change in mRNA levels of *ZEB1* and *SNAI2* in HKe3 ER:HRAS V12 cells with the indicated treatments. mRNA levels normalized to *GAPDH* in control cells were used to set the baseline value at unity. Data are mean ± s.d. *n* = 3 samples per group. ** *P* < 0.01. *** *P* < 0.001. (**D**) Protein expression of CDH1, SNAI2, SQSTM1, RELA and ATG12–ATG5 in PANC1 cells with the indicated treatments. TUBB was used as a loading control. (**E**) Fold change in mRNA levels of *CDH1* and *SNAI2* in PANC1 cells with the indicated siRNA transfections. mRNA levels normalized to *GAPDH* in control cells were used to set the baseline value at unity. Data are the means of 2 independent experiments carried out in duplicate.

**Figure S7.** Accumulation of SQSTM1 correlates with negative membrane CDH1 expression in pancreatic adenocarcinoma. (**A**) The relationship between *SQSTM1* mRNA and protein expression in the TCGA pancreatic adenocarcinoma Provisional dataset was analyzed (Mann-Whitney U-test: *P* = 0.2138). Low SQSTM1 (Z-score < -0.3), *n* = 48 and high SQSTM1 (Z-score > 0.3), *n* = 43. (**B**) No significant relationship between *SQSTM1* mRNA level and mRNA level of *CDH1* in the TCGA pancreatic adenocarcinoma Provisional dataset (Spearman’s R = 0.15, *n* = 179, *P* = 0.0503). (**C**) Representative human pancreatic adenocarcinomas and adjacent normal pancreatic tissues show the presence or absence of SQSTM1 accumulation. Scale bar: 100 μm.
